# Partner separation rescues pair bond-induced decreases in hypothalamic oxytocin neural densities

**DOI:** 10.1038/s41598-023-32076-8

**Published:** 2023-03-24

**Authors:** Brandon A. Fricker, Venezia C. Roshko, Jinrun Jiang, Aubrey M. Kelly

**Affiliations:** Department of Psychology, Emory University, 36 Eagle Row, Atlanta, GA 30322 USA

**Keywords:** Social behaviour, Social neuroscience, Neuroscience

## Abstract

Studies in prairie voles (*Microtus ochrogaster*) have shown that although formation of the pair bond is accompanied by a suite of behavioral changes, a bond between two voles can dissolve and individuals can form new pair bonds with other conspecifics. However, the neural mechanisms underlying this behavioral flexibility have not been well-studied. Here we examine plasticity of nonapeptide, vasopressin (VP) and oxytocin (OT), neuronal populations in relation to bonding and the dissolution of bonds. Using adult male and female prairie voles, animals were either pair bonded, co-housed with a same-sex sibling, separated from their pair bond partner, or separated from their sibling. We examined neural densities of VP and OT cell groups and observed plasticity in the nonapeptide populations of the paraventricular nucleus of the hypothalamus (PVN). Voles that were pair bonded had fewer PVN OT neurons, suggesting that PVN OT neural densities decrease with pair bonding, but increase and return to a pre-pair bonded baseline after the dissolution of a pair bond. Our findings suggest that the PVN nonapeptide cell groups are particularly plastic in adulthood, providing a mechanism by which voles can exhibit context-appropriate behavior related to bond status.

## Introduction

The pair bond is a hallmark of socially monogamous species where individuals form strong, stable mating relationships with a conspecific. Pairs cohabitate, reproduce, and engage in biparental care towards offspring across several reproductive cycles. Although social monogamy is common among humans and is observed in various avian species^1^, it is uncommon in mammals, with only an estimated 9% of mammalian species forming pair bonds^2^. Studies exploring pair bonding most frequently use the prairie vole, (*Microtus ochrogaster*), a rodent that robustly pairs with novel, opposite-sex conspecifics and has been used rather successfully for the systematic exploration of behavior associated with social monogamy (e.g. Aragona & Wang and Winslow et al.^3,4^) and relevant underlying neural and genetic mechanisms^4–7^. Indeed, prairie voles are valued as an organism with a mating structure that is representative of human romantic bonds^3,8^. Most recently, prairie voles have also proven to be excellent models for the behavioral and neural effects of loss and separation from a partner^9,10^.

The formation of a pair bond in prairie voles is associated with modifications to the brain and behavior. For example, compared to unpaired voles, paired voles display higher rates of selective aggression towards novel, opposite-sex conspecifics^11,12^. Additionally, both bonded male and female voles highly prefer to affiliate and huddle with their partner over novel, opposite-sex conspecifics in a long-term partner preference test^13,14^, and preferentially mate with their bonded partner over other conspecifics^15^.

The nonapeptides vasopressin (VP) and oxytocin (OT) modulate an array of behaviors ranging from affiliation and pair bonding to aggression and anxiety^16–18^. The contributions of VP, OT, and their receptors, to pair bonding have been studied for several decades (e.g.^5,10,12,14,19–23^), arguably with a greater focus on nonapeptide receptor-mediated behavior^14,19,24^. After the identification of species differences in OTR distributions in monogamous and polygamous voles^25,26^, researchers began to inhibit or facilitate pair bonding via neural manipulations. For example, blocking V1aR activation prior to cohabitation and mating prevents male prairie voles from displaying a partner preference^4^, whereas OTR knockdown in the nucleus accumbens disrupts partner preference formation in female prairie voles^20^. Numerous other studies have further demonstrated the importance of nonapeptide receptors in pair bonding^5,25,27–29^.

There has been considerably less insight obtained into the importance of and changes within the source populations for the nonapeptides, likely because receptor densities are highly variable across and within species whereas less variability is typically observed in nonapeptide-producing neuronal populations^30–32^. The formation of a pair bond, however, increases VP mRNA in the bed nucleus of the stria terminalis (BST)^33^. Dissolution of a pair bond, meanwhile, has also been linked to changes within nonapeptide producing neuronal populations. Sun et al.^34^ found that the number of VP and OT producing neurons within the paraventricular nucleus of the hypothalamus (PVN) and only OT producing neurons within the supraoptic nucleus of the hypothalamus were higher in prairie voles separated from their partner than those still paired. Notably, this study only compared partnered and separated male voles and did not test whether neural differences were specific to pair bonding. In other words, it is possible that the differences observed in paired and separated males may have been due to social isolation rather than specifically to the dissolution of the pair bond. Additionally, whether the observed changes in VP/OT cell number also occur in female prairie voles and if nonapeptide neural densities differ between virgin and pair bonded voles, to our knowledge, remain unknown.

The changes in social behaviors associated with forming a pair bond, along with accompanying modifications to the nonapeptide system, suggest that the act of pair bonding may permanently change the brain and behavior. However, a recent study demonstrated that male prairie voles can be separated from their partners and bonded to a new female conspecific 10 times^21^. Given that prairie voles exhibit selective aggression after pairing to help maintain the pair bond, this behavior and underlying mechanisms must change after separation from a partner to allow a new bond to be formed in the future. Yet it remains unknown whether the ability to return to a state where another pair bond may be formed, coined “Rewritable fidelity”^21^, is accomplished through reverting the neuronal nonapeptide populations back to a baseline from before a bond formed, or via some other mechanism. More broadly, despite a vast literature on the influences of the early life environment on developmental differences in nonapeptide neuron and receptor densities^22,35–37^, we know little about just how plastic nonapeptide neuronal populations are across adulthood (but see Grippo et al.^38^). It is unclear whether nonapeptide neuronal densities are as fluid as nonapeptide receptor densities, which have been shown to fluctuate with bonding status^12^.

Here we compared behavior and VP/OT neuronal densities between not only male and female pair bonded and separated (i.e., previously pair bonded) prairie voles but also with sexually naïve paired siblings and isolated siblings in order to test the specificity of nonapeptide neuronal changes associated with pair bonding. We examined VP and OT cell groups of the PVN, BST, and anterior hypothalamus (AH), all of which may undergo changes associated with pair bonding given their involvement in affiliation, anxiety, and aggression^11,17,39–41^. Because (a) Sun et al.^34^ observed a difference in PVN OT between paired and separated male prairie voles and (b) prairie voles can break a pair bond and exhibit a decrease in bonding-induced selective aggression thus allowing for the formation a new pair bond^9,21^, we hypothesized that PVN OT and AH VP neuronal densities would be different for pair bonded animals compared to those housed with a sibling, separated from a pair bond partner, and separated from a sibling. This would suggest that adult nonapeptide neuronal populations exhibit plasticity, potentially to enable state-dependent titration of peptide to promote context-appropriate behavior, such as promoting aggression to maintain a pair bond but inhibiting aggression if that bond dissolves, therefore enabling the formation of a new bond. Alternatively, if nonapeptide cell numbers are more similar between pair bonded and sibling-housed voles than voles separated from a pair bond partner or sibling, this would suggest that neural changes are likely due to the stress of social isolation rather than properties specific to a pair bond.

## Results

### Experimental design

In the present study, adult male and female prairie voles were randomly assigned to 1 of 4 conditions: (1) pair bonded with an opposite-sex conspecific (Pair Bond), (2) pair bonded and subsequently separated from their partner (Separated Pair), (3) sexually naïve and housed with a same-sex sibling (Sibling), or (4) sexually naïve and housed with a same-sex sibling, but subsequently separated from their sibling (Separated Sibling). 2 weeks after co-housing, all subjects were run through a modified resident-intruder test. For subjects that were pair bonded, animals also underwent testing in a partner preference test. Animals in the Pair Bond and Sibling conditions were then perfused, and brains were collected for histological analysis. For animals in the Separated Pair and Separated Sibling conditions, subjects were then separated from their partner or sibling and remained in isolation for 30 days. Subjects in the Separated Pair condition were later tested again in a modified resident-intruder and partner preference test, whereas animals in the Separated Sibling condition only underwent a modified resident-intruder test. Brains were then collected for histology for the remaining subjects. Here, we aimed to examine whether the number of nonapeptide-producing neurons in several brain regions associated with pair bonding induced-changes in prairie vole behavior display properties that may allow for rewritable fidelity.

### Pair bonding decreases PVN OT cell numbers but separation from a partner restores densities to pre-pairing levels

**Figure 1:**
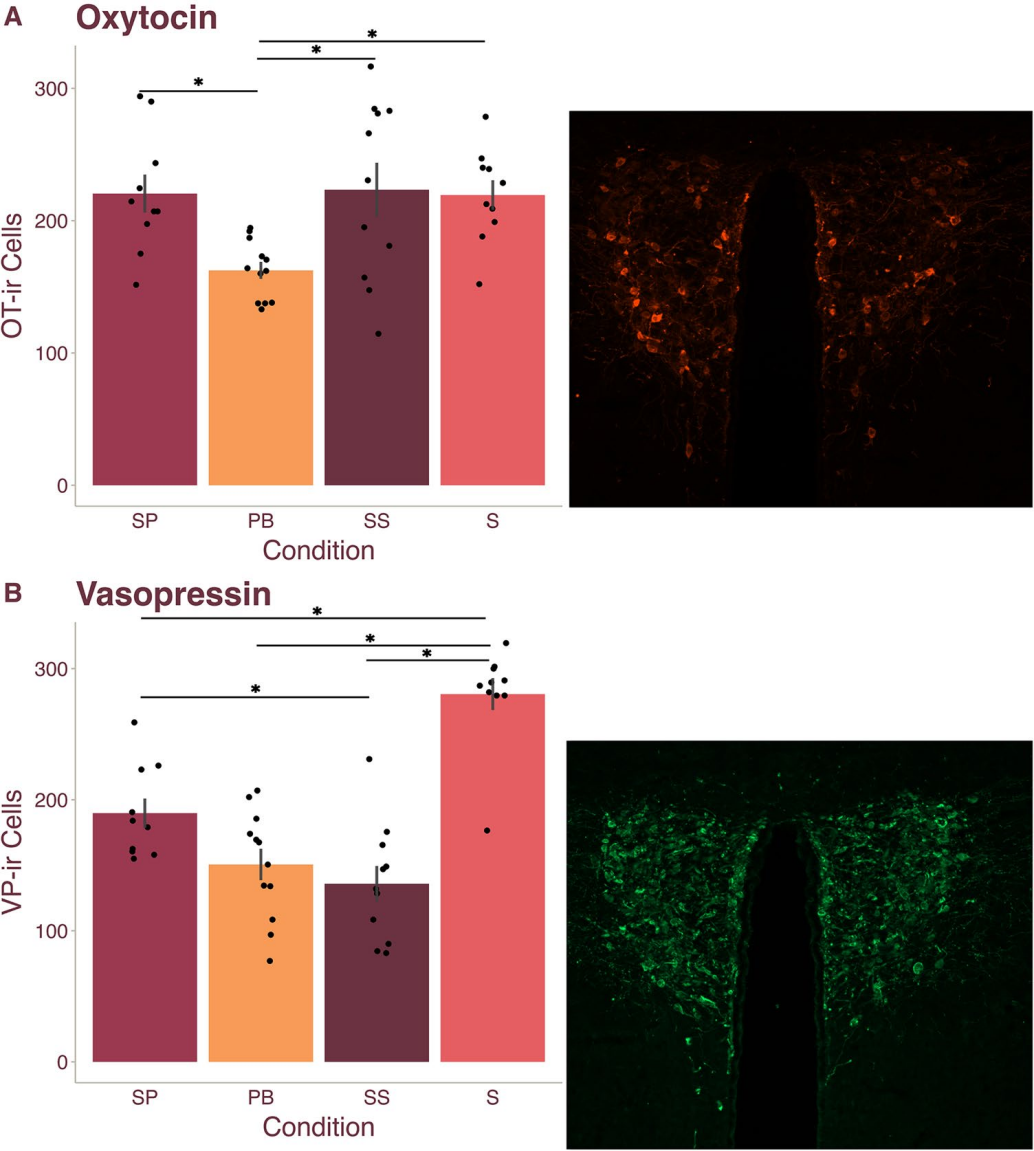
PVN oxytocin and vasopressin densities differ based on bond status. Legend: *SP* Separated Pair, *PB* Pair Bond, *SS* Separated Sibling, *S* Sibling. (**A**) Male and female prairie vole mean ($\pm$ SEM) PVN oxytocin (OT) cell numbers (left) with a representative image of the PVN OT neuronal population (right). PVN OT neural densities in voles in the PB condition were significantly lower than densities of voles in all other conditions. (**B**) Male and female prairie vole mean ($\pm$ SEM) PVN VP cell numbers (left) with a representative image of the PVNVP neuronal population (right). PVN VP neural densities in voles in the SP condition were significantly higher than those in the SS condition, and PVN VP neural densities in voles in the SB condition were significantly higher than densities in voles in all other conditions. Dots represent individual data points. *Indicated *p* < 0.05.

We first compared OT cell numbers within the PVN between the Pair Bond, Separated Pair, Sibling, and Separated Sibling conditions. A GLM analysis with Condition and Sex as fixed factors revealed that PVN OT differed between conditions (F_(3, 35)_ = 5.224, *p* = 0.004), but not across sex (F_(1,35)_ = 0.291, *p* = 0.593). The interaction between Condition and Sex was also not significant (F_(3, 35)_ = 2.337, *p* = 0.091). Bonferroni corrected post hoc comparisons first revealed that the Separated Pair (M = 216.229) condition had significantly more OT immunoreactive (-ir) cells than the Pair Bond (M = 162.417, *p* = 0.019, d = 1.220) condition (Fig. 1A). These results reflect the findings of Sun et al.^34^ where the Pair Bond condition had significantly fewer OT-ir cells in the PVN compared to a Separated Pair condition. However, our post hoc analysis further revealed that the Separated Pair, Sibling (M = 219.833), and Separated Sibling (M = 226.033) conditions did not differ (all *p* = 1.000), while the Pair Bond condition had significantly fewer OT-ir cells than all three other conditions (all *p* < 0.019, all d > 1.220) (Fig. 1A). Although the results in Sun et al.^34^ were interpreted as partner separation inducing an increase in PVN OT, by adding control conditions using non-pair bonded animals, we reveal an alternative conclusion: pair bonding appears to lower PVN OT neuronal densities, while breaking a pair bond appears to return PVN OT cell numbers to pre-pair bond levels.

### Pair bonding and separation decrease PVN vasopressin cell numbers

To determine whether the number of VP-ir neurons changes based on forming or breaking sexual bonds, we ran a GLM with Condition and Sex as fixed factors. VP-ir neurons differed across conditions (F_(3, 35)_ = 28.033, *p* < 0.001), but not sex (F_(1, 35)_ = 3.274, *p* = 0.079); additionally, we did not observe an interaction between Condition and Sex (F_(3, 35)_ = 0.804, *p* = 0.500). Post hoc analysis revealed that the Separated Pair (M = 190.129) condition did not differ from the Pair Bond condition (M = 150.583, *p* = 0.161) but was significantly higher than the Separated Sibling (M = 138.142, *p* = 0.032, d = 1.290) condition and significantly lower than the Sibling (M = 283.417, *p* < 0.001, d = 2.381) condition (Fig. 1B). Interestingly, the Sibling condition was significantly higher than all other conditions (all *p* < 0.001, all d > 2.314) (Fig. 1B). Together, these results suggest that the number of VP-ir neurons may decrease in response to impactful life events such as bonding and separation (i.e., social isolation), potentially reflecting global changes associated with stress response.

### OT and VP within the BST and AH do not exhibit plasticity in relation to bonding or separation

**Figure 2:**
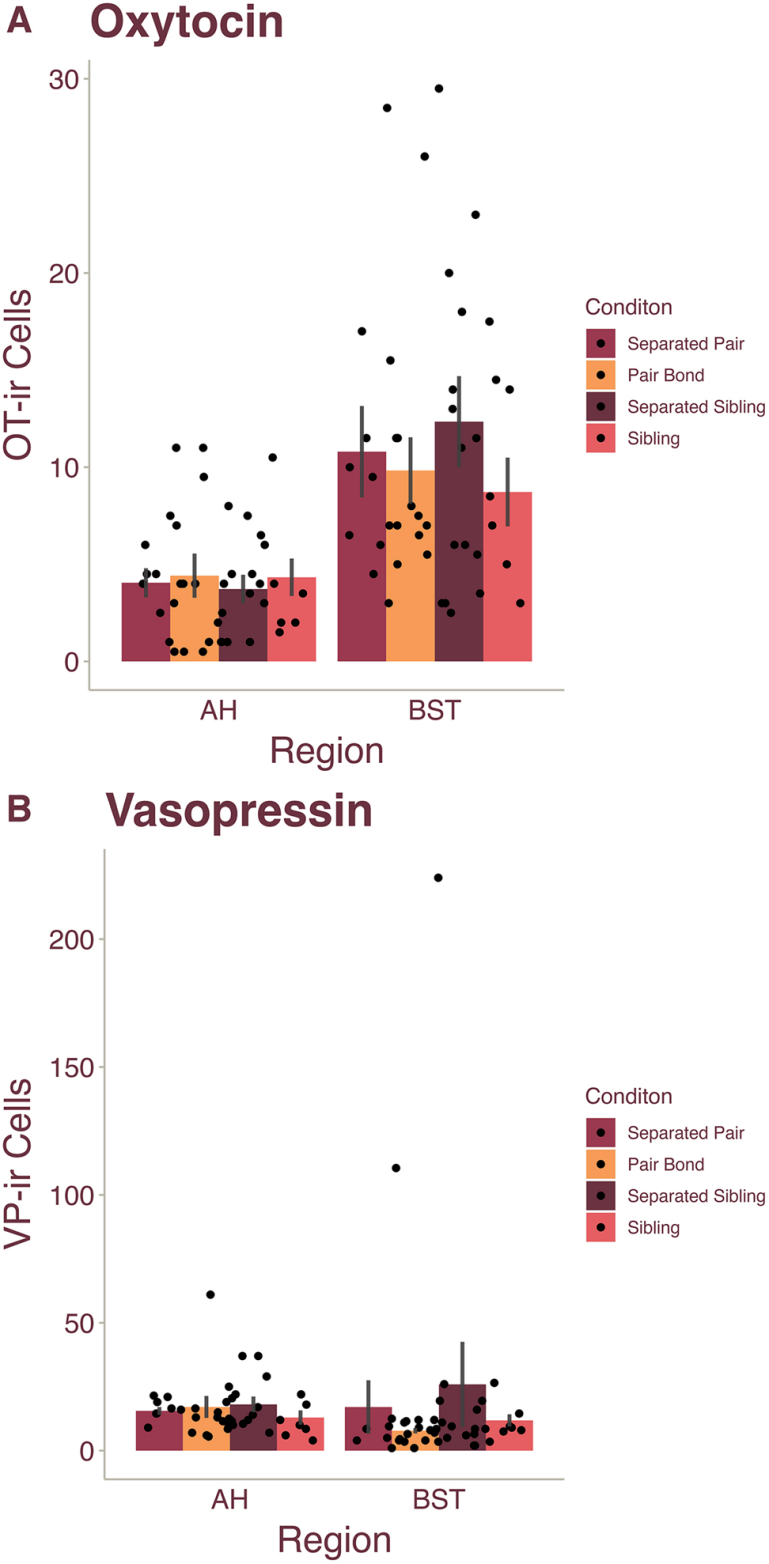
Oxytocin and vasopressin neural densities did not differ in the AH or BST based bond status. Male and female prairie vole mean ($\pm$ SEM) (**A**) oxytocin (OT) and (**B**) vasopressin (VP) cell numbers within the AH and BST. Dots represent individual data points.

Next, we examined OT and VP cell numbers in the BST and AH between the Pair Bond, Separated Pair, Sibling, and Separated Sibling conditions. For OT-ir cell counts in both brain regions, we ran GLM analyses with Condition and Sex as fixed factors. In the BST, there were no differences across Condition (F_(3,36)_ = 0.679, *p* = 0.570), Sex (F_(1, 36)_ = 1.49, *p* = 0.701), or the condition-sex interaction (F_(3, 36)_ = 1.365, *p* = 0.269) (Fig. 2A). Similarly, for the AH OT neuronal population there were no significant differences across Condition (F_(3, 34)_ = 0.108), p = 0.955) or Sex (F_(1, 34)_ = 0.337, *p* = 0.565); additionally, we observed no significant interactions (F_(3, 34)_ = 1.189, *p* = 0.329) (Fig. 2A). VP-ir cell numbers were also analyzed using a GLM and revealed similar results. In the BST, we found no significant differences across Condition (F_(3, 36)_ = 0.475, *p* = 0.702) or Sex (F_(1, 36)_ = 0.234, *p* = 0.632) and no interactions (F_(3, 36)_ = 0.769, *p* = 0.519) (Fig. 2B). Lastly, for the AH VP cell group, we observed no significant differences across Condition (F_(3, 34)_ = 0.694, *p* = 0.620) or Sex (F_(1, 34)_ = 0.010, *p* = 0.919), as well as no significant interactions (F_(3, 34)_ = 1.312, *p* = 0.286) (Fig. 2B). Together, our results suggest that the nonapeptide populations of the BST and AH are less plastic than those of the PVN in relation to pair bonding and bond dissolution.

### The preference for a pair bond partner persists after separation

**Figure 3:**
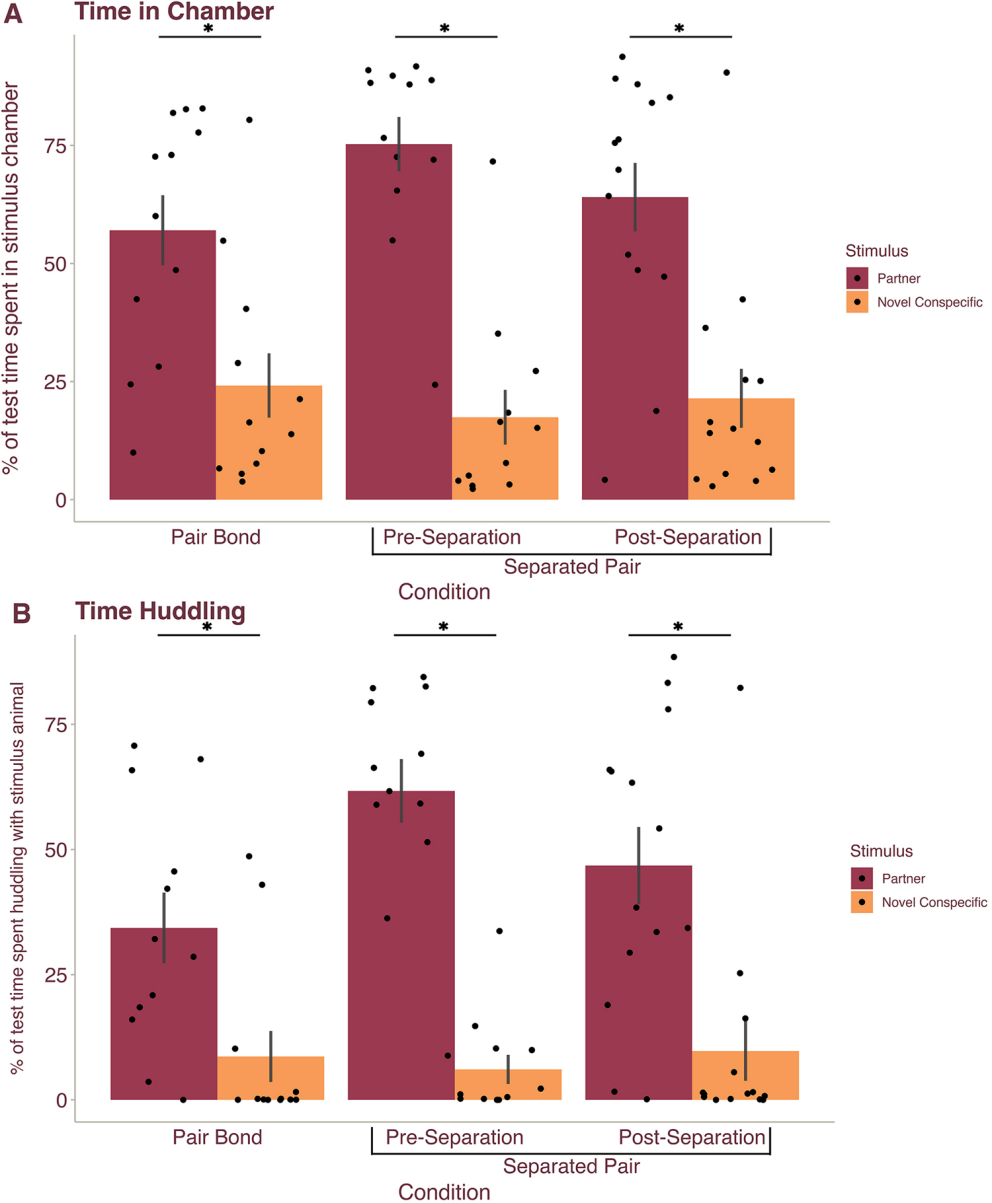
A partner preference persists after separation. Data from the partner preference test (PPT) in voles in the Pair Bond (PB) condition and from the PPT tests pre- and post- separation in voles in the Separated Pair (SP) condition. Mean ($\pm$ SEM) percentage of time male and female prairie voles spent in the (**A**) stimulus chamber and (**B**) engaged in huddling with partner (magenta) and a novel, opposite-sex conspecific (orange). All subjects spent a significantly larger percentage of test time in the chamber and huddling with their partner over the novel, opposite-sex conspecific. Dots represent individual data points.

To confirm that a pair bond has been formed, it is field standard to conduct a partner preference test in which the subject is placed in a multi-chambered apparatus and the time spent huddling with the partner vs. an opposite-sex stranger is recorded^9,21,34,42^. To confirm that voles in the Pair Bond and Separated Pair conditions had indeed formed pair bonds, we tested subjects in a partner preference test (e.g. prior to separation for the Separated Pair condition). A GLM analysis with Condition (Pair Bond or Separated Pair), Stimulus (novel, opposite-sex conspecific or partner), and Sex as fixed factors revealed no significant differences across the sexes but did reveal a main effect of Stimulus, with significant differences in the percentage of test time spent in the partner and novel conspecific chambers (F_(1, 44)_ = 28.18, *p* < 0.001) and the percentage of test time spent huddling with the partner and novel conspecific (F_(1, 44)_ = 21.397, *p* < 0.001). Post hoc analyses confirmed that male and female voles spent a larger percentage of the test in the partner chamber (M = 60.54, *p* < 0.001, d = 1.47) (Fig. 3A) and a greater percentage of test time spent huddling with the partner (M = 40.57, *p* < 0.001, d = 1.28) than the novel, opposite-sex conspecific (M = 22.805, M = 9.202) (Fig. 3B).

To determine if the preference for a pair bond partner dissolved after 30 days of partner separation, subjects in the Separated Pair condition underwent a second partner preference test post-separation from the partner. A repeated measures GLM analysis with Time (pre- versus post- separation) as a repeated measure and Stimulus (novel, opposite-sex conspecific or partner), and Sex as fixed factors yielded a main effect of Stimulus, with the percentage of test time spent in the partner versus novel conspecific chamber (F_(1, 20)_ = 64.606, *p* < 0.001) and the percentage of test time spent huddling with the partner or novel conspecific (F_(1, 20)_ = 69.209, *p* < 0.001) significantly varying. Male and female voles spent a larger percentage of the test in the partner chamber (M = 72.598, *p* < 0.001, d = 3.28) (Fig. 3A) and huddling with their partner (M = 57.7356, *p* < 0.001, d = 3.39) (Fig. 3B). Surprisingly, the analysis showed no effect of Time such that the percentage of test time in the stimulus chambers and huddling with stimuli did not significantly differ between the pre-separation and post-separation partner preference tests (all *p* < 0.219). Further, we found no effect of Sex and no interactions between fixed factors (all *p* > 0.173). Together this suggests that the strength of the preference for a pair bond partner did not change after 30 days of separation. Thus, our analyses confirm that the voles in our study successfully bonded with their partners but continued to show a preference for their former partner even after a prolonged period of separation.

### Selective aggression was present only in females in a modified resident-intruder paradigm

A hallmark of pair bonding in prairie voles is the onset of selective aggression toward novel, opposite-sex conspecifics after pairing^12^ To confirm that pair bonded voles exhibited selective aggression, to test whether partner separation resulted in a decrease in aggression toward a potential mate, and to test the specificity of selective aggression to pair bonding (i.e., as opposed to after cohabitating with a sibling), we ran all subjects through modified resident-intruder tests where the subject was placed in a new cage for 20 min prior to the introduction of a novel, opposite-sex intruder. The Pair Bond and Sibling conditions underwent a single modified resident intruder test whereas the Separated Pair and Separated Sibling conditions underwent a resident intruder test before and after 30 days of separation.

**Figure 4:**
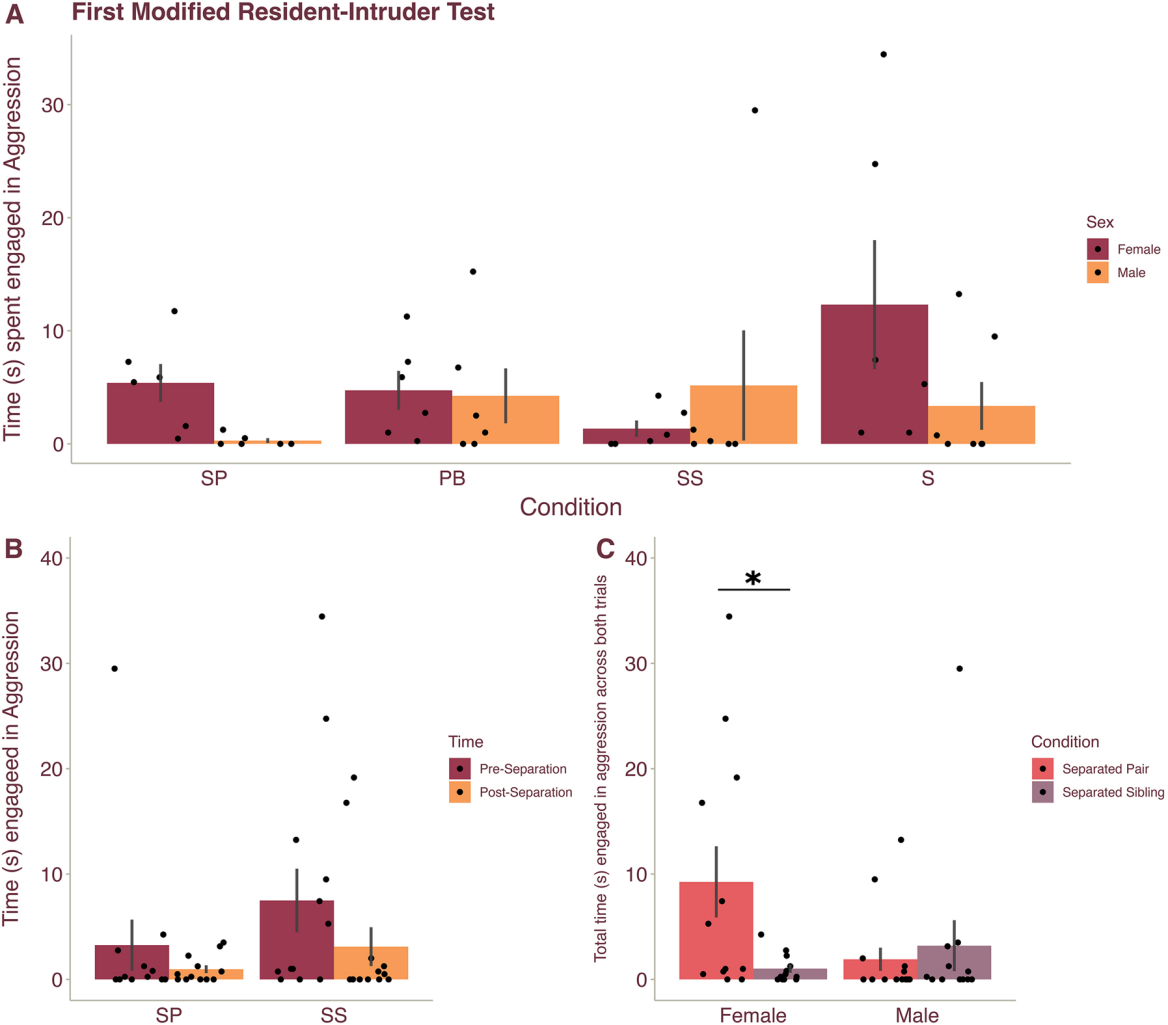
Selective aggression was observed only in female voles. Legend: *SP* Separated Pair, *PB* Pair Bond, *SS* Separated Sibling, *S* Sibling. (**A**) Mean ($\pm$ SEM) time male (orange) and female (magenta) prairie voles were engaged in aggression with a novel, opposite-sex conspecific during the initial modified resident-intruder test. (**B**) Mean ($\pm$ SEM) time male and female prairie voles were engaged in aggression in the SP and SS conditions before (magenta) and after (orange) separation from their partner/sibling. (**C**) Mean ($\pm$ SEM) time male and female prairie voles were engaged in aggression collapsed across Time (i.e., tests pre- and post-separation) for voles in the SP (pink) and SS (purple) conditions. Females in the SP condition exhibited more aggression toward a novel, opposite-sex conspecific compared to females in the SS condition. Dots represent individual data points. *Indicates *p* < 0.05.

We first compared aggression in the first modified resident intruder test across all subjects using a GLM with Condition and Sex as fixed factors. We expected to observe no difference in aggression between animals in the Pair Bond and Separated Pair conditions, as well as between the Sibling and Separated Sibling conditions, because no animals had been separated from their partner/sibling at the time of the first modified resident intruder test. However, we sought to determine whether pair bonded animals exhibit greater aggression toward novel, opposite-sex conspecifics compared to sibling housed animals. Interestingly, there was no main effect of Condition (F_(3, 41)_ = 1.185, *p* = 0.327), such that aggression did not differ across conditions—even between pair bonded and sibling housed voles (Fig. 4A). We also found no effect of Sex on aggression and no interaction between Sex and Condition (all *p* > 0.172).

We then compared aggression before and after separation for the Separated Pair and Separated Sibling conditions using a repeated measures GLM with Time (pre- versus post- separation) as a repeated measure and Condition (Separated Pair versus Separated Sibling) and Sex as fixed factors. Analyses revealed that within subjects, there were no effects of Time, Sex, or Condition, and no interactions including Time (all *p* > 0.094) (Fig. 4B). However, across subjects we observed a significant interaction between Condition and Sex for the amount of time subjects engaged in aggression (F_(1,21)_ = 5.803, *p* = 0.025). Posthoc analyses showed that Separated Pair females (M = 9.257) were significantly more aggressive than female subjects in the Separated Sibling condition (M = 1.027, *p* = 0.009, d = 1.18) (Fig. 4C), suggesting that, with more datapoints per individual (i.e., data from two modified resident-intruder tests), we were able to observe that pair bonding did indeed induce selective aggression in females.

## Discussion

Our findings suggest robust plasticity of peptide production within the PVN OT neuronal population based on the bonding state of an adult prairie vole. We observed that 2 weeks of pair bonding resulted in a decrease of OT-ir cell numbers within the PVN, but dissolution of the pair bond after 30 days of separation returned PVN OT neural densities to pre-bonding levels. We further observed plasticity of the PVN VP cell group, however, changes in neural densities were not specific to pair bond status. Conversely, the VP and OT neuronal populations of the BST and AH exhibited more rigidity such that cell densities did not vary based on bonding state. Curiously, despite the neural indicator of a “return to baseline” for the PVN OT cell group, both male and female voles continued to display strong preferences for their original pair bond partner even after 30 days of separation.

### The role of PVN OT in rewritable fidelity

Exhibiting flexibility within neuronal populations can allow for significant changes in function and behavior that promote adaptation to a new environment or context^43^. At a systems level, without being able to “reset” and either form a new bond or readily mate with novel conspecifics, voles may significantly reduce their reproductive rate after the death of a partner. Indeed, field studies have shown that although female prairie voles that are separated from their partner do not often form a new bond, they continue to reproduce^44^; thus, it is possible that the flexibility in nonapeptide neuronal populations observed in the present study may facilitate openness to mating with novel conspecifics in the absence of a partner. Thus, rewritable fidelity conveys significant advantages to socially monogamous rodents, and various mechanisms have likely evolved to enable flexibility in bonding behaviors. For example, the transcription profile of the nucleus accumbens in prairie voles changes after bonding, but this change erodes over 4 weeks after separation^10^. Similarly, after 10 pair bond experiences, both the OTR and V1aR receptor densities were mostly unchanged relative to the first bond^21^. Here we add to this growing literature on rewritable fidelity with the findings that the PVN OT cell group exhibits lower neural densities in pair bonded animals compared to voles that were co-housed with siblings or had been separated from their partner or sibling. Our results suggest that while pair bonding may induce a decrease in PVN OT, this cell group returns to a pre-bond baseline after separation. This return to baseline is not driven by social isolation, as the Sibling and Separated Sibling conditions, which act as controls for social isolation, did not differ. Notably, other studies have also demonstrated flexibility in the PVN OT cell group of adults; stress-decreases the number of OT-producing neurons in the PVN in a rat post-partum depression model^45^. It is perhaps not surprising that the PVN OT neuronal population may contribute toward a vole’s ability to break and form new bonds given the dynamic involvement of the cell group in physiology and behavior. The PVN has been implicated in managing stress^46^ and feeding behavior^47^, anxiety and aggression^48,49^, and maternal defense^50^. It is this involvement in an array of systems that may lead the PVN OT cell group to be highly sensitive to shifts in the environment, allowing an animal to respond in a context-appropriate manner.

Our findings also suggest that the PVN VP cell group exhibits plasticity within adulthood, however, differences in neural densities were not specific to pair bonding. Indeed, a previous study showed that social isolation lowers PVN VP cell numbers in adult prairie voles^51^. Although pair bonded animals in our study exhibited fewer PVN VP cells than animals co-housed with a sibling, voles that were separated from their bonds and socially isolated also exhibited fewer PVN VP cells. Thus, it is possible that PVN VP neural densities may decrease to help maintain a pair bond and could potentially return to a pre-bond baseline to allow for a new bond to be formed, but such a finding may have been masked by an isolation-induced decrease in PVN VP. Alternatively, although PVN VP may be plastic, it is feasible that PVN OT plays a greater role in mediating pair bond-specific context-appropriate behavior.

### Interpreting cell numbers: a consideration of stress and anxiety

The interpretation of differences in OT-ir and VP-ir neuronal numbers is challenging. On one hand, a greater number of OT cells may indicate that less OT is being released due to lower demand for the peptide. In this scenario, voles in the Pair Bond condition may have fewer PVN OT-ir cells because the peptide is being rapidly released and not stored in the cell body due to a high demand for the peptide. However, an alternative explanation is that more OT cells may suggest greater peptide production, such that more OT is required to meet environmental/behavioral demands. From this perspective, fewer OT neurons in animals in the Pair Bond condition may suggest that less PVN OT needs to be produced after a vole is pair bonded. Because OT has previously been suggested as an anxiolytic in rodents^17^, our data may therefore suggest that being pair bonded is less stressful than being separated from a bond or being paired with a sibling (i.e., when a vole is potentially in a state of readiness to find and form a pair bond) and, thus, less PVN OT is required. Supporting this interpretation, loss of a pair bond partner has been shown to induce depressive-like behavior^52^ and anxiety-like behavior^53^, so it is feasible that pair bonded voles in our study were less stressed and/or anxious. Further, successfully bonding with a partner may reduce stress because it provides opportunities for mating (i.e., increased fitness) as well as consolation from a partner to reduce anxiety-like behaviors^54^.

### A partner is highly memorable

In our study, male and female voles that were separated from their pair bond partners exhibited a preference to huddle with their previous partners over novel, opposite-sex individuals even after 30 days of separation. Although Sun et al.^34^ found that a partner preference dissolved in males previously paired with ovariectomized females after 4 weeks, other studies have demonstrated that prairie voles can remember and prefer to affiliate with their partners up to prolonged periods of separation (up to 4 weeks)^9,10^. Interestingly, after 20 days of separation, male and female voles exhibit behavior towards a sibling as if they were a novel, same-sex individual, suggesting that social memories of siblings and nonsexual bonds may not be particularly salient^55^. Alternatively, a growing number of studies suggest that a pair bond partner may be especially engrained in social memory. Indeed, paired, but not unpaired, male voles showed successful social recognition of novel females in a habituation/dishabituation style task, suggesting that pairing may influence memory processes^56^. Pairing itself may improve social memory, given that even while bonded to a new partner, 4 weeks of separation is required for the second partner to be preferred over the first^9^. However, because we did not assess the bond strength between same-sex siblings in the Sibling and Separated Sibling conditions, we can only assess the effects of social isolation and sexual experience, not sexual vs. asexual bonds. The neural mechanisms underlying this heightened memory and continued preference are unclear but will undoubtably further our understanding of neuronal plasticity and the influences of pair bonding on cognition and the brain.

### Sex differences in selective aggression

In our modified resident-intruder task, we did not observe a consistent and/or robust difference in aggression towards an opposite-sex novel conspecific across subjects of varying bond status. We observed heightened aggression only when comparing pair bonded vs. sibling housed females, and only in females that were in the Separated conditions and had undergone 2 modified resident-intruder tests. The repeated measure of Time was not significant in this analysis, suggesting that more datapoints per subject were needed to observe Condition differences in aggression. It is possible that we did not consistently observe the selective aggression characteristic of pair bonded prairie voles in all subjects because our modified resident-intruder tasks took place in a novel cage after only 20 min of subject habitation because both partners living in a single cage were subjects and tested simultaneously. However, previous studies in prairie voles have shown that aggressive behavior does not differ during interactions with a novel *same*-sex conspecific in the homecage or a neutral cage^57^. Our findings suggest that males may be less aggressive, and potentially less choosey, than female voles in interactions with a new potential mate. Consistent with this, female prairie voles show preferences for more affiliative males as well as males with larger anogenital distances^58,59^. Importantly, when our dataset is broken up by condition and sex, our sample sizes are relatively small. Future studies should explore the possibility of female choosey-ness with more robust and sex-effect focused sample sizes.

Together, our results suggest that the PVN nonapeptide cell groups are plastic in adulthood, and that plasticity within the PVN OT cell group may be especially important for exhibiting context-appropriate behavior such that a vole is able to maintain an established pair bond but be receptive to new mates if not currently pair bonded.

## Methods and materials

### Animals

The study complied with the ARRIVE guidelines. 27 male and 27 female adult prairie voles (post-natal day (PND) 71-162), with sex defined based on external genitalia, were obtained from our breeding colony; breeders were from the captive-bred colony of Dr. Alex Ophir (Cornell University). All animals were group housed in a standard rat cage (40.64 × 20.32 × 20.32 cm) lined with Sani-Chips bedding. Animals were provided with nesting material and enrichment and were able to obtain food (Lab Rabbit Diet HF #5326, LabDiet) and water ad libitum. The voles were kept on a 14L:10D light cycle with an ambient temperature of 24 ± 2 °C. Sample sizes for behavioral testing:6 females and 7 males for the Separated Pair condition, 6 females and 6 males for the Separated Sibling condition, 6 females and 6 males for the Pair Bond condition, and 6 female and 6 male voles for the Sibling condition. Due to unexpected mortalities and tissue damage or loss, data were not available for neural analyses from animals in the following conditions: 2 females and 1 male in the Separated Pair condition, 1 female in the Separated Sibling condition, and 2 females in the Sibling condition. Please note that sample sizes were not “topped up” because this experiment was conducted prior to, and up until, the onset of the Covid-19 pandemic. The experiment was cut short and ended when the university shut down due to the pandemic. At that time, our prairie vole animal colony was permanently terminated, and we no longer work with this species in the lab. All voles in the Sibling and Separated Sibling condition were sexually naïve. All experimental procedures were in agreement with animal welfare laws in the United States. All procedures were approved by the Institutional Animal Care and Use Committee of Emory University (PROTO201900094). All experimental procedures were in accordance with relevant institutional guidelines and regulations which also adhered to animal welfare laws in the United States.

### Pair bond and sibling condition testing timelines

For the Pair Bond and Sibling conditions, all voles underwent a modified resident-intruder test 12 days after being paired with a partner. The Pair Bond condition voles then underwent a partner preference test 13 or 14 days after pairing, with the day of testing for each individual in a pair counterbalanced. After testing was complete, all subjects were perfused (i.e., 14 days after pairing). See Fig. S1A for a testing timeline.

### Separated pair and separated sibling condition testing timelines

For the Separated Pair and Separated Sibling conditions, all voles underwent the same testing schedule as the Pair Bond and Sibling conditions up to day 14. On day 14, instead of perfusion, all subjects were separated from their partner or sibling and were single-housed for 30 days. All subjects then underwent a second modified resident-intruder test on day 42. For the Separated Pair condition, both voles of a pair underwent a partner preference test as previously described on either day 43 or 48. To limit the chance of refamiliarizing with their partner, five days separated these tests, and the order of testing was counterbalanced. All subjects for the Separated Pair and Separated Sibling conditions were euthanized by isoflurane overdose and were transcardially perfused on day 49. See Fig. S1B for a testing timeline.

### Modified resident-intruder test

All subjects underwent a modified resident-intruder test on day 12 where subjects were allowed to acclimate in a clean cage for 20 min before a novel, same-sex intruder was introduced. The subject and stimulus animal were able to interact for 6 min before separated and returned to their home cage. The first five minutes of the modified resident-intruder test were scored for prosocial (allogrooming, positive side-by-side contact, huddling, head investigation, flank investigation, and rear investigation), aggressive (biting, chasing, lunging, pinning, rearing, and aggressive side-by-side contact) and non-overt (all remaining time of scored recording) behaviors.

### Partner preference test

For conditions where a bond was formed (Pair Bond and Separated Bond), both voles of a pair underwent a partner preference test on separate days, with the sex of the vole tested on each day counterbalanced. Subjects were placed in the middle chamber of a three-chamber testing apparatus (76.2 × 25.4 × 30.48 cm) With their partner tethered in one end chamber and a novel, opposite-sex conspecific placed in the other. The chamber location for the partner and stimulus animal were counterbalanced across subjects. The subject was allowed to move into any chamber and interact with the tethered partner or stimulus conspecific for 3 h before being returned to their home cage. For the entire 3 h, the time spent in the middle room, novel stimulus room, and partner room was scored along with time engaged in prosocial behavior, huddling behavior, mating behavior and aggression with either the partner or novel conspecific. The Sibling and Separated Sibling conditions did not undergo a partner preference test as the tests were used to confirm the formation and potential dissolution of bonds within pair bonded conditions.

### Histology and immunohistochemistry

Subjects were euthanized by isoflurane overdose and were transcardially perfused with 0.1 M phosphate buffer saline (PBS) followed by 4% paraformaldehyde. Brains were extracted, post-fixed overnight in 4% paraformaldehyde, and underwent cryoprotection in 30% sucrose dissolved in PBS for 48 h. Brains were frozen in Tissue-Tek O.C.T. compound and stored at − 80 °C before sectioning coronally at 40 µm using a Leica cryostat, with every third section saved for use in the present study. Tissue sections were immunofluorescently stained for OT and VP following previously established protocols^60^. Tissue was rinsed 5× for 10 min in 0.1 M PBS (pH 7.4), incubated for 1 h in a blocking solution (PBS + 10% normal donkey serum + 0.03% Triton-X-100) to prevent non-specific binding, and then incubated for approximately 48 h in primary antibodies diluted in PBS containing 5% normal donkey serum + 0.03% Triton-X-100. Primary antibodies used were guinea pig anti-VP (1:1000; BMA Biomedicals, Switzerland; Catalog # T-5048; RRID:AB_518680) and mouse anti-OT (3:1000; Millipore, Billerica, MA; Catalog #MAB 5296; RRID:AB_2157626). The primary incubation was then followed by two 30 min rinses in PBS. Tissue incubated for 1 h in a biotinylated donkey anti-guinea pig secondary antibody (8:1000; Jackson Immunoresearch, West Grove, PA), was rinsed twice for 15 min in PBS, and incubated for 2 h at room temperature in streptavidin conjugated to Alexa Fluor 488 (3:1000) and donkey anti-mouse secondary conjugated to Alexa Fluor 594 (5:1000). All secondary antibodies were diluted in PBS containing 5% normal donkey serum + 0.03% Trion-X-100. Alexa Fluor conjugates were obtained from ThermoFisher Scientific (Waltham, MA). Following two 30 min rinses in PBS, sections were mounted on microscope slides and cover-slipped with Prolong Gold antifade containing a DAPI nuclear stain (ThermoFisher Scientific).

### Neural quantification

Photomicrographs were obtained using a Zeiss AxioImager II microscope fitted with an apotome. 10× photomicrographs of the PVN, BST, and AH were obtained, and we quantified the total number of OT-ir and VP-ir cells across 2 tissue sections per region. For the BST and AH, these were sequential sections, but for the PVN, to include both the rostral and caudal PVN we imaged tissue sections that were roughly 120 µm apart. FIJI^61^ was used to create standard ROIs for all dorsal and ventral LS images, and a cell profiler^62^ pipeline was created to automatically count fluorescent cells. Cell counts were averaged across tissue sections for analyses.

### Statistical analysis

Behavioral measurements for each test were analyzed using SPSS 28 (IBM Analytics). The use of parametric or non-parametric tests was based on the distribution of the data and Shapiro-Wilks tests. The tests used include general linear models (GLM) with Condition and Sex as fixed factors, as well as repeated measure GLMs with Condition and Sex as fixed factors and Time (pre or post-separation) as a repeated measure. All post-hoc pairwise comparisons were adjusted using the Bonferroni correction. The tests used for specific analyses are detailed in the Results. Outliers for each individual test were three standard deviations outside the mean and were removed from analyses. Effect sizes for normally distributed data were calculated and reported as Cohen’s d.

## Supplementary Information

Supplementary Figure S1.

## Data Availability

The datasets analyzed for the current study are available from the corresponding author upon request.

## References

[1] Carter CS, Perkeybile AM (2018). The monogamy paradox: What do love and sex have to do with it?. Front. Ecol. Evol..

[2] Lukas D, Clutton-Brock TH (2013). The evolution of social monogamy in mammals. Science.

[3] Aragona BJ, Wang Z (2004). The prairie vole (*Microtus ochrogaster*): An animal model for behavioral neuroendocrine research on pair bonding. ILAR J..

[4] Winslow JT, Hastings N, Carter CS, Harbaugh CR, Insel TR (1993). A role for central vasopressin in pair bonding in monogamous prairie voles. Nature.

[5] Ophir AG, Gessel A, Zheng D-J, Phelps SM (2012). Oxytocin receptor density is associated with male mating tactics and social monogamy. Horm. Behav..

[6] Walum H, Young LJ (2018). The neural mechanisms and circuitry of the pair bond. Nat. Rev. Neurosci..

[7] Young KA, Gobrogge KL, Liu Y, Wang Z (2011). The neurobiology of pair bonding: Insights from a socially monogamous rodent. Front. Neuroendocrinol..

[8] Kenkel WM, Gustison ML, Beery AK (2021). A neuroscientist’s guide to the vole. Curr. Protoc..

[9] Harbert KJ, Pellegrini M, Gordon KM, Donaldson ZR (2020). How prior pair-bonding experience affects future bonding behavior in monogamous prairie voles. Horm. Behav..

[10] Sadino JM (2022). Prolonged partner separation erodes nucleus accumbens transcriptional signatures of pair bonding in male prairie voles. Elife.

[11] Gobrogge KL, Liu Y, Jia X, Wang Z (2007). Anterior hypothalamic neural activation and neurochemical associations with aggression in pair-bonded male prairie voles. J. Comp. Neurol..

[12] Gobrogge KL, Liu Y, Young LJ, Wang Z (2009). Anterior hypothalamic vasopressin regulates pair-bonding and drug-induced aggression in a monogamous rodent. Proc. Natl. Acad. Sci..

[13] Beery AK, Christensen JD, Lee NS, Blandino KL (2018). Specificity in sociality: Mice and prairie voles exhibit different patterns of peer affiliation. Front. Behav. Neurosci..

[14] Williams JR, Catania KC, Carter CS (1992). Development of partner preferences in female prairie voles (Microtus ochrogaster): The role of social and sexual experience. Horm. Behav..

[15] Carter CS, Getz LL (1993). Monogamy and the prairie vole. Sci. Am..

[16] Goodson JL, Thompson RR (2010). Nonapeptide mechanisms of social cognition, behavior and species-specific social systems. Curr. Opin. Neurobiol..

[17] Neumann ID, Landgraf R (2012). Balance of brain oxytocin and vasopressin: Implications for anxiety, depression, and social behaviors. Trends Neurosci..

[18] Rigney N, de Vries GJ, Petrulis A, Young LJ (2022). Oxytocin, vasopressin, and social behavior: From neural circuits to clinical opportunities. Endocrinology.

[19] Carter CS, Williams JR, Witt DM, Insel TR (1992). Oxytocin and social bonding. Ann. N. Y. Acad. Sci..

[20] Keebaugh AC, Barrett CE, Laprairie JL, Jenkins JJ, Young LJ (2015). RNAi knockdown of oxytocin receptor in the nucleus accumbens inhibits social attachment and parental care in monogamous female prairie voles. Soc. Neurosci..

[21] Kenkel WM, Perkeybile AM, Yee JR, Carter CS (2019). Rewritable fidelity: How repeated pairings and age influence subsequent pair-bond formation in male prairie voles. Horm. Behav..

[22] Rogers FD, Freeman SM, Anderson M, Palumbo MC, Bales KL (2021). Compositional variation in early-life parenting structures alters oxytocin and vasopressin 1a receptor development in prairie voles (Microtus ochrogaster). J. Neuroendocrinol..

[23] Williams JR, Insel TR, Harbaugh CR, Carter CS (1994). Oxytocin administered centrally facilitates formation of a partner preference in female prairie voles (*Microtus ochrogaster*). J. Neuroendocrinol..

[24] Williams JR, Carter CS, Insel T (1992). Partner preference development in female prairie voles is facilitated by mating or the central infusion of oxytocin. Ann. N. Y. Acad. Sci..

[25] Insel TR, Shapiro LE (1992). Oxytocin receptor distribution reflects social organization in monogamous and polygamous voles. Proc. Natl. Acad. Sci..

[26] Young LJ, Huot B, Nilsen R, Wang Z, Insel TR (1996). Species differences in central oxytocin receptor gene expression: Comparative analysis of promoter sequences. J. Neuroendocrinol..

[27] Hirota Y (2020). Oxytocin receptor antagonist reverses the blunting effect of pair bonding on fear learning in monogamous prairie voles. Horm. Behav..

[28] Johnson ZV (2016). Central oxytocin receptors mediate mating-induced partner preferences and enhance correlated activation across forebrain nuclei in male prairie voles. Horm. Behav..

[29] Ross HE (2009). Variation in oxytocin receptor density in the nucleus accumbens has differential effects on affiliative behaviors in monogamous and polygamous voles. J. Neurosci..

[30] Freeman AR, Aulino EA, Caldwell HK, Ophir AG (2020). Comparison of the distribution of oxytocin and vasopressin 1a receptors in rodents reveals conserved and derived patterns of nonapeptide evolution. J. Neuroendocrinol..

[31] Kelly AM, Goodson JL (2014). Social functions of individual vasopressin–oxytocin cell groups in vertebrates: What do we really know?. Front. Neuroendocrinol..

[32] King LB, Walum H, Inoue K, Eyrich NW, Young LJ (2016). Variation in the oxytocin receptor gene predicts brain region-specific expression and social attachment. Biol. Psychiatr..

[33] Wang Z, Smith W, Major DE, De Vries GJ (1994). Sex and species differences in the effects of cohabitation on vasopressin messenger RNA expression in the bed nucleus of the stria terminalis in prairie voles (Microtus ochrogaster) and meadow voles (Microtus pennsylvanicus). Brain Res..

[34] Sun P, Smith AS, Lei K, Liu Y, Wang Z (2014). Breaking bonds in male prairie vole: Long-term effects on emotional and social behavior, physiology, and neurochemistry. Behav. Brain Res..

[35] Kelly AM, Ong JY, Witmer RA, Ophir AG (2020). Paternal deprivation impairs social behavior putatively via epigenetic modification to lateral septum vasopressin receptor. Sci. Adv..

[36] Perkeybile AM (2019). Early nurture epigenetically tunes the oxytocin receptor. Psychoneuroendocrinology.

[37] Prounis GS, Thomas K, Ophir AG (2018). Developmental trajectories and influences of environmental complexity on oxytocin receptor and vasopressin 1A receptor expression in male and female prairie voles. J. Comp. Neurol..

[38] Grippo AJ (2007). Social isolation induces behavioral and neuroendocrine disturbances relevant to depression in female and male prairie voles. Psychoneuroendocrinology.

[39] Carcea I (2021). Oxytocin neurons enable social transmission of maternal behaviour. Nature.

[40] Duque-Wilckens N (2020). Extrahypothalamic oxytocin neurons drive stress-induced social vigilance and avoidance. Proc. Natl. Acad. Sci..

[41] Kelly AM, Goodson JL (2014). Hypothalamic oxytocin and vasopressin neurons exert sex-specific effects on pair bonding, gregariousness, and aggression in finches. Proc. Natl. Acad. Sci..

[42] Lim MM (2004). Enhanced partner preference in a promiscuous species by manipulating the expression of a single gene. Nature.

[43] Kelly AM, Vitousek MN (2017). Dynamic modulation of sociality and aggression: an examination of plasticity within endocrine and neuroendocrine systems. Philos. Trans. R. Soc. B Biol. Sci..

[44] Thomas SA, Wolff JO (2004). Pair bonding and “the widow effect” in female prairie voles. Behav. Proc..

[45] Wang T (2018). Injection of oxytocin into paraventricular nucleus reverses depressive-like behaviors in the postpartum depression rat model. Behav. Brain Res..

[46] Grippo AJ, Cushing BS, Carter CS (2007). Depression-like behavior and stressor-induced neuroendocrine activation in female prairie voles exposed to chronic social isolation. Psychosom. Med..

[47] Iwasaki Y (2019). Relay of peripheral oxytocin to central oxytocin neurons via vagal afferents for regulating feeding. Biochem. Biophys. Res. Commun..

[48] Bosch OJ, Meddle SL, Beiderbeck DI, Douglas AJ, Neumann ID (2005). Brain oxytocin correlates with maternal aggression: Link to anxiety. J. Neurosci..

[49] He Z (2019). Increased anxiety and decreased sociability induced by paternal deprivation involve the PVN-PrL OTergic pathway. Elife.

[50] Bosch OJ, Krömer SA, Brunton PJ, Neumann ID (2004). Release of oxytocin in the hypothalamic paraventricular nucleus, but not central amygdala or lateral septum in lactating residents and virgin intruders during maternal defence. Neuroscience.

[51] Ruscio MG, Sweeny T, Hazelton J, Suppatkul P, Sue Carter C (2007). Social environment regulates corticotropin releasing factor, corticosterone and vasopressin in juvenile prairie voles. Horm. Behav..

[52] Bosch OJ, Nair HP, Ahern TH, Neumann ID, Young LJ (2009). The CRF system mediates increased passive stress-coping behavior following the loss of a bonded partner in a monogamous rodent. Neuropsychopharmacol.

[53] Osako Y (2018). Partner loss in monogamous rodents: Modulation of pain and emotional behavior in male prairie voles. Psychosom Med.

[54] Burkett JP (2016). Oxytocin-dependent consolation behavior in rodents. Science.

[55] Paz y miñatno G, Tang-martínez Z (1999). Effects of isolation on sibling recognition in prairie voles, *Microtus ochrogaster*. Anim. Behav..

[56] Blocker TD, Ophir AG (2015). Social recognition in paired, but not single, male prairie voles. Anim. Behav..

[57] Harper SJ, Batzli GO (1997). Are staged dyadic encounters useful for studying aggressive behaviour of arvicoline rodents?. Can. J. Zool..

[58] Ophir AG, Crino OL, Wilkerson QC, Wolff JO, Phelps SM (2008). Female-directed aggression predicts paternal behavior, but female prairie voles prefer affiliative males to paternal males. BBE.

[59] Ophir AG, del Barco-Trillo J (2007). Anogenital distance predicts female choice and male potency in prairie voles. Physiol. Behav..

[60] Kelly AM, Fricker BA, Wallace KJ (2022). Protocol for multiplex fluorescent immunohistochemistry in free-floating rodent brain tissues. STAR Protoc..

[61] Schindelin J (2012). Fiji: An open-source platform for biological-image analysis. Nat. Methods.

[62] Stirling DR (2021). Cell Profiler 4: Improvements in speed, utility and usability. BMC Bioinf..

