# Supplementary material for: Partner separation rescues pair bond-induced decreases in hypothalamic oxytocin neural densities
Source: Sci Rep. 2023 Mar 24;13:4835. doi: 10.1038/s41598-023-32076-8 (PMC10037388; doi:10.1038/s41598-023-32076-8)
Supplement: Supplementary file 1 — Supplementary Figure S1. [file 41598_2023_32076_MOESM1_ESM.docx]

**Supplementary Materials**

**
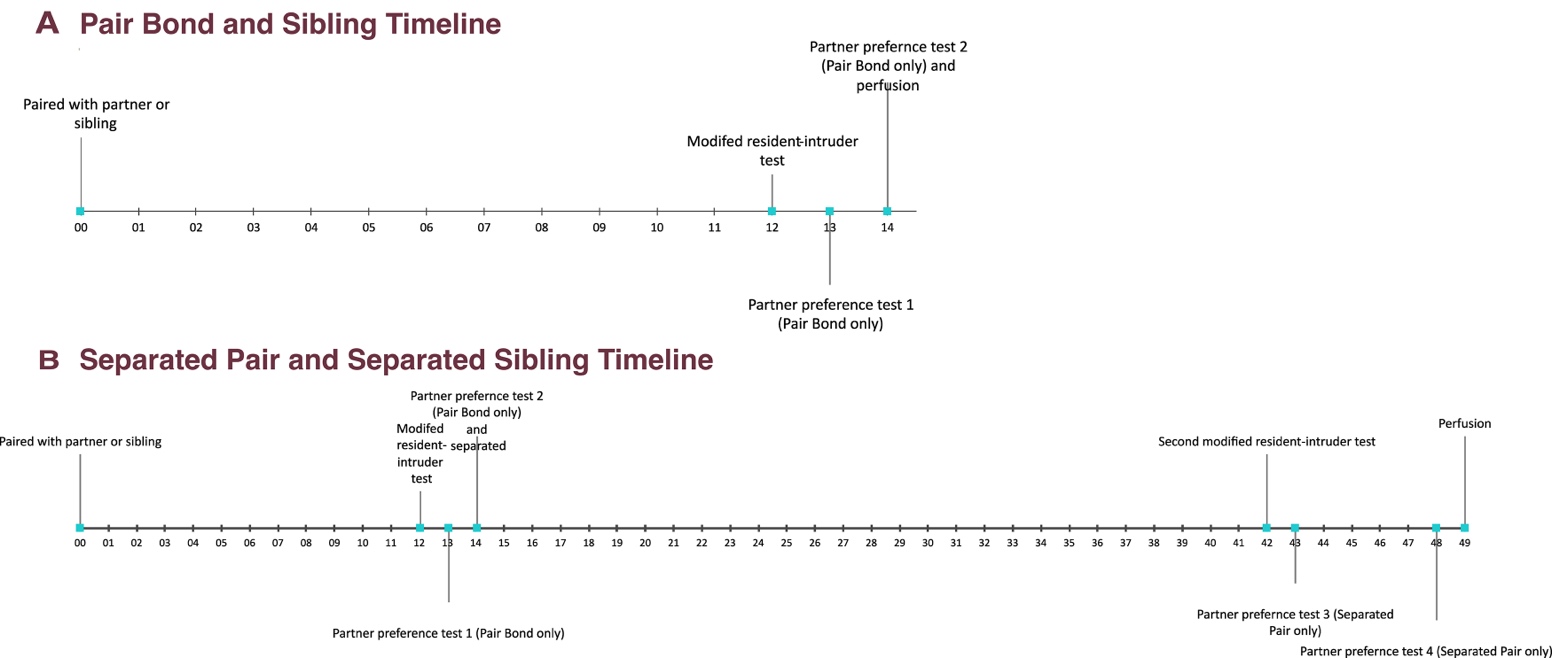
**

**Fig. S1. Behavioral testing timelines. (A)** Subjects in the Pair Bond or Sibling conditions were paired with a partner or sibling on Day 1. On Day 12, all subjects underwent a modified resident-intruder test. Because both animals in a pair bond were subjects, a partner preference test was conducted for 1 animal in the pair on Day 13 and the other animal in the pair on Day 14. All subjects were perfused on Day 14 after behavioral testing. **(B)** Subjects in the Separated Pair and Separated Sibling conditions were tested as described above through Day 14. However, after testing on Day 14, pair bonds and cohabitating siblings were separated, and subjects were singly housed. On Day 42, all subjects underwent a second modified resident-intruder test. For subjects in the Separated Pair condition, animals underwent a second partner preference test on Day 43 or Day 48. All subjects were perfused on Day 49.
